# Mutational status of K- *ras* and *TP53* genes in primary sarcomas of the heart

**DOI:** 10.1054/bjoc.1999.1060

**Published:** 2000-02-21

**Authors:** J M Garcia, R Gonzalez, J M Silva, G Dominguez, I S Vegazo, C Gamallo, M Provencio, P España, F Bonilla

**Affiliations:** Departments of Medical Oncology and Pathology, Clinica Puerta de Hierro, Madrid, 28035, Spain; Department of Pathology, Hospital La Paz, Madrid, 28034, Spain

**Keywords:** cardiac sarcomas, K-*ras* mutations, *TP53* mutations

## Abstract

We investigated three patients with cardiac angiosarcomas and two with cardiac rhabdomyosarcomas, all for mutations at exons 5, 6, 7 and 8 of the *p53* gene and at exon 1 of K- *ras*. No point mutations were observed in the *p53* gene in any of the five cases; however, at exon 1 of K- *ras*, three patients (60%) presented the same mutation at the first base of codon 13 (G to A transition). Interestingly, this mutation was detected in both rhabdomyosarcoma and angiosarcoma histologic sarcoma types. © 2000 Cancer Research Campaign


British Journal of Cancer (2000) **82**(6), 1183–1185
© 2000 Cancer Research Campaign
Article no. bjoc.1999.1060, available online at http://www.idealibrary.com on IDEAL®

### Mutational status of K-*ras* and *TP53* genes in primary sarcomas of the heart

**JM Garcia[1], R Gonzalez[1], JM Silva[1], G Dominguez[1], IS Vegazo[2], C Gamallo[3], M Provencio[1], P España[1] and F Bonilla[1]**

Departments of [1]Medical Oncology and [2]Pathology, Clinica Puerta de Hierro, 28035-Madrid, Spain; [3]Department of Pathology, Hospital La Paz, 28034-Madrid, Spain

**Summary** We investigated three patients with cardiac angiosarcomas and two with cardiac rhabdomyosarcomas, all for mutations at exons 5, 6, 7 and 8 of the *p53* gene and at exon 1 of K-*ras*. No point mutations were observed in the *p53* gene in any of the five cases; however, at exon 1 of K-*ras*, three patients (60%) presented the same mutation at the first base of codon 13 (G to A transition). Interestingly, this mutation was detected in both rhabdomyosarcoma and angiosarcoma histologic sarcoma types. © 2000 Cancer Research Campaign

**Keywords:** cardiac sarcomas; K-*ras* mutations; *TP53* mutations

Primary tumours of the heart have an incidence of only 0.001–0.28% (McAllister and Fenoglio, 1978). Of these, approximately 25% are sarcomas occurring in adults (Silverman, 1980). Angiosarcomas and rhabdomyosarcomas are the most frequent histological subtypes (Putnam et al, 1991). In general, the survival of these patients is short (Molina et al, 1990; Putnam et al, 1991; Burke et al, 1992) despite complete surgical resection by heart transplantation (Crespo et al, 1993).

The molecular characterization of primary cardiac sarcomas with respect to genetic alterations in *p53* and K-*ras* genes has not been widely studies. However, there is some evidence of the presence of *p53* and K-*ras* gene alterations in sarcomas located in other organs (Muligan et al, 1990). A number of reports dealing with the occurrence of *p53* gene mutations in sarcomas show it to be a frequent event, especially in the rhabdomyosarcoma histologic subtype (Felix et al, 1992; Wurl et al, 1996; Kusufuka et al, 1997). Specifically, Naka et al (1997) reported two cases of *p53* gene mutations in four patients with primary cardiac angiosarcomas.

Different mutation rates of *ras* genes have been detected in sarcomas. K-*ras* mutations in 26% of hepatic angiosarcomas (Przygodzki et al, 1997), point H-*ras* mutations in 33% of rhabdomyosarcomas and malignant fibrous histiocytomas (Wilke et al, 1993), and N-*ras* and K-*ras* mutations in 35% of rhabdomyosarcomas (Stratton et al, 1989). However, genetic studies of heart sarcomas have not been reported.

We investigated the mutational status of *p53* and K-*ras* genes in primary cardiac sarcomas in a small number of patients.

*Received 24 May 1999*
*Revised 6 October 1999*
*Accepted 24 November 1999*

*Correspondence to:* F Bonilla, Molecular Genetics Unit, Clinica de Trabajo, Avd. de la Reina Victoria 21, 28003-Madrid, Spain

### MATERIALS AND METHODS

### Tumour samples and DNA extraction

We obtained tumour specimens from seven patients in two Madrid hospitals. Five samples corresponded to sarcomas (three angiosarcomas and two rhabdomyosarcomas) with two additional samples corresponding to rhabdomyomas being taken from infants who died of multiple cardiac rhabdomyomas 3 and 7 days after birth. All the histological specimens were fixed in 10% formalin and routinely processed by paraffin-embedding. Histologic confirmation of the diagnosis was performed prior to the molecular study. DNA was extracted from formalin-fixed, paraffin-embedded tissues using chelating resin. Paraffin blocks without samples were used as negative controls for each polymerase chain reaction (PCR) analysis throughout the procedure.

### Mutational study of the *p53* gene

To establish the presence of point mutations in the conserved exons of TP53, polymerase chain reaction/single-strand conformation polymorphism (PCR-SSCP) analysis was performed. Exons 5, 6, 7 and 8 of the *p53* gene were amplified. PCR was performed under standard conditions in a 25-μl volume containing 2-μl (100 ng) tumour DNA template; 2.5-μl of 10 × PCR buffer and 1.5 U of Ampli *Taq* Gold (Perkin-Elmer, Roche Molecular Systems Inc., Branchburg, NJ, USA); 200 μM deoxynucleotide triphosphate (dNTP) mix; 0.6 μM of each primer; and various concentrations of magnesium chloride ($MgCl_2$) depending on the primer and distilled water ($H_2O$) needed to reach the total volume. For PCR amplification, the samples underwent 40 cycles at 94°C for 1 min, followed by subjection to different annealing temperatures depending on the primer, and 70°C for 1 min. The amplified products were denatured by mixing with 15 μl of denaturing stop solution that contained 98% formamide, 10 ml $l^{-1}$ edathamil (pH 8.0), 0.02% xylene cyanol and 0.02% bromophenol blue, being heated to 95°C for 5 min and rapidly cooled on ice. Electrophoresis was

**Table 1** Molecular and clinicopathologic patients characteristics

| Age | Sex | Histology | Chamber | Treatment | Postsurg. ChT | Survival (months) | Status | *p53* gene Mut | K-*ras* Mut |
|---|---|---|---|---|---|---|---|---|---|
| 54 | F | Angio | RA | No specific | No | 5 | D | No | No |
| 27 | M | Rhabdo | LA | Resection | ADM | 7 | D | No | Yes |
| 31 | M | Angio | RA | Transpl | ADM | 10 | D | No | Yes |
| 36 | M | Angio | RA | Transpl | ADM | 8 | D | No | Yes |
| 52 | F | Rhabdo | LV | Transpl | ADM | 45 | D | No | No |

F, female; M, male; Angio, angiosarcoma; Rhabdo, rhabdomyosarcoma; RA, right atrium, LA, left atrium: LV, left ventricle; Transpl, heart transplantation; D, dead; Mut, mutations; ADM, doxorubicin; ChT, chemotherapy.

run on non-denaturing 8–12% polyacrylamide gels for 12–15 h at 250 V. The allelic band intensity on the gels was detected by non-radioisotopic means using a commercially available silver staining method (Oto et al, 1993). All specimens that showed a differential band at SSCP were amplified to obtain templates for DNA sequencing. These amplifications were independent to those used for SSCP analysis. Amplified DNA fragments were purified from 0.9% agarose gels using the Geneclean Kit (Bio-101, Inc., La Jolla, CA, USA) and used for direct DNA sequencing by the ddNTP method with the Sequenase Kit (United States Biochemical Corp. Cleveland, OH, USA).

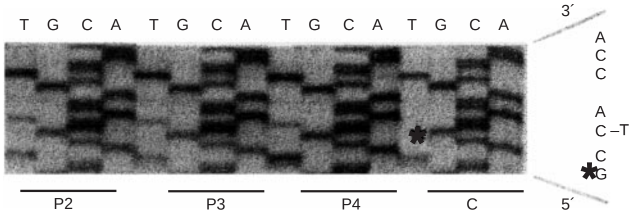

**Figure 1** Sequencing analysis of patients P2, P3 and P4 shows a nucleotide change (C to T) in the reverse sequence that corresponds to a transition (G to A) of the K-*ras* gene. This transition at codon 13 causes an amino acid change (Gly-Ser). The change was not observed in the DNA of the control case (C)

### Mutational analysis of K-*ras* gene

PCR amplification of K-*ras* gene, using 100 ng genomic DNA as template, was carried out in a 25-µl reaction volume with a final concentration of 1 × PCR buffer and 1.5 units of Ampli *Taq* DNA polymerase (Perkin-Elmer, Roche Molecular Systems Inc., Branchburg, NJ, USA); 200 µM dNTP mix; 0.6 µM of each primer; 2.5 mM $MgCl_2$ and distilled $H_2O$ to reach the total volumes. For amplification, each sample was denatured at 94°C for 5 min and subjected to 35 cycles (94°C for 30 s, 58°C for 40 s and 72°C for 30 s, followed by a final 7-min extension at 72°C). The amplified products were mixed with 20 µl denaturing stop solution that contained 98% formamide, 10 mml $l^{-1}$ edathamil (pH 8.0), 0.02% xylene cyanol and 0.02% bromophenol blue, heated to 95°C for 5 min and rapidly cooled on ice. The samples were electrophoresed on non-denaturing 12% polyacrylamide gels at 250 V overnight at room temperature. Products were visualized by non-radioisotopic means using a commercially available silver staining method (Oto et al, 1993). Primers used for amplification of exon 1 of K-*ras*, which contains codons 12 and 13, were: 5′-GACTGAATATAAACTTGTGGTAGT and 5′-CTATTGTTGGATCATATTCGTCC. All specimens, with and without differential bands at SSCP, were sequenced following the same method as used in the sequencing process of *p53* gene.

### RESULTS

The clinicopathologic characteristics of the patients and treatments administered are listed in Table 1.

The five cardiac sarcomas were screened for mutations by PCR-SSCP and direct sequencing at exons 5, 6, 7 and 8 of the *p53* gene and exon 1 of the K-*ras* gene. SSCP analysis of the exons of the *p53* gene did not reveal any cases of bands with altered electrophoretic mobility. Considering that SSCP sensitivity is low under certain circumstances (Hayashi, 1991) direct sequence analysis for exons 5, 6, 7 and 8 of the *p53* gene in each case were performed and no point mutations were detected. The first exon of K-*ras* was checked by SSCP in all lesions and three sarcomas showed a mobility shift; however, to avoid false-negatives, direct sequence analysis was performed in all cases. Mutations at codon 13 of K-*ras* were present in all three cases that displayed changes in mobility. G to A transition at the first base of codon 13, which resulted in one amino acid substitution (Gly-13 to Ser) (Figure 1), was detected in two angiosarcomas and one rhabdomyosarcoma.

Although our series does not permit a statistical study due to its small size, in terms of clinicopathologic features, the three patients with K-*ras* mutations were under 50 years of age (mean 31 years) and the two patients without K-*ras* mutations were over 50. Moreover, it is noteworthy that the same K-*ras* mutation was present in both rhabdomyosarcoma and angiosarcoma.

### DISCUSSION

The rare occurrence of primary cardiac sarcomas may explain the almost complete absence of molecular studies involving the molecular status of *p53* and K-*ras* genes in these tumours. To the best of our knowledge, there have been no reports dealing with mutations in the *ras* gene family, and there is only one report of a study examining *p53* gene mutations in cardiac angiosarcomas with point mutations being found in 50% of cases (Naka et al, 1997). No point mutations in the *p53* gene were detected in the cases we present here, suggesting that it is difficult to draw conclusions about *p53* inactivation in these rare tumours from small series.

However, the current study shows that K-*ras* gene mutations could be relatively frequent in these rare tumours. Of the five cases studied, we found a mutation at the first position of codon 13 (Gly-13 to Ser) in three sarcomas, two angiosarcomas and one rhabdomyosarcoma, while no mutations were observed in the two cardiac rhabdomyomas assessed. Although the implication of the

 *© 2000 Cancer Research Campaign*

*ras* oncogene family in the development of mesenchymal tumours was reported long ago (Stratton et al, 1989) there were no studies available concerning the presence of these point mutations in sarcomas derived from heart tissue.

As was mentioned above, the clinicopathologic features of our patients were similar to those reported elsewhere with respect to age, tumour location and histological type. In our series, it is interesting to note that the same mutation was observed in both a heart rhabdomyosarcoma and an angiosarcoma.

In conclusion, we report a small series of heart sarcomas subjected to a specific molecular study that shows the presence of K-*ras* mutations at codon 13. Nevertheless, there were no differential clinical characteristics except for the patients harbouring these mutations being younger and predilection for the male sex.

### ACKNOWLEDGEMENTS

We are indebted to Mrs M Messman and Mr M Hadley-Adams for the revision and preparation of the manuscript. This work was supported by grants from the Fundación Caja Madrid and Bristol-Myers, S.A.

### REFERENCES

Burke AP, Cowan D and Virmani R (1992) Primary sarcomas of the heart. *Cancer* **69**: 387–395

Crespo MG, Pulpon LA, Pradas G, Serrano S, Segovia J, Vegazo I, Salas C, España P, Silva L and Burgos R (1993) Heart transplantation for cardiac angiosarcoma: should its indication be questioned? *J Heart Lung Transplant* **12**: 527–530

Felix CA, Kappel CC, Mitsudomi T, Nau MM, Tsokos M, Crouch GD, Nisen PD, Winick NJ and Helman LJ (1992) Frequency and diversity of p53 mutations in childhood rhabdomyosarcoma. *Cancer Res* **52**: 2243–2247

Hayashi K (1991) PCR-SSCP: a simple and sensitive method for detection of mutations in the genomic DNA. *PCR Methods Appl* **1**: 34–38

Kusafuka T, Fukuzawa M, Oue T, Komoto Y, Yoneda A and Okada A (1997) Mutation analysis of p53 gene in childhood malignant solid tumors. *J Pediatr Surg* **32**: 1175–1180

McAllister HA and Fenoglio JJ (1978) Tumors of the cardiovascular system. In: *Atlas of Tumor Pathology*, vol. 15, pp. 73–119. Washington DC: Armed Forces Institute of Pathology

Molina JE, Edwards JE and Ward HB (1990) Primary cardiac tumors: experience at the University of Minnesota. *J Thorac Cardiovasc Surg* **38**: 183–191

Mulligan LM, Matlashewski GJ, Scrable HJ and Cavenee WK (1990) Mechanism of p53 loss in human sarcomas. *Proc Natl Acad Sci USA* **87**: 5863–5867

Naka M, Tomita Y, Nakanishi H, Araki N, Hongyo T, Ochi T and Aozasa K (1997) Mutations of *p53* tumor-suppressor gene in angiosarcoma. *Int J Cancer* **71**: 952–955

Oto M, Miyake S and Yuasa Y (1993) Optimization of nonradioisotopic single strand conformation polymorphism analysis with a conventional minislab gel electrophoresis apparatus. *Anal Biochem* **213**: 19–22

Przygodzki RM, Finkelstein SD, Keohavong P, Zhu D, Bakker A, Swalsky PA, Soini Y, Ishak KG and Bennett WP (1997) Sporadic and thorotrast-induced angiosarcomas of the liver manifest frequent and multiple point mutations in K-ras-2. *Lab Invest* **76**: 153–159

Putnam JB, Sweeney MS, Colon R, Lanza LA, Frazier OH and Cooley D (1991) Primary cardiac sarcomas. *Ann Thorac Surg* **51**: 906–910

Silverman NA (1980) Primary cardiac tumors. *Ann Surg* **191**: 127–138

Stratton MR, Fisher C, Gusterson BA and Cooper CS (1989) Detection of point mutations in N-ras and K-ras genes of human embryonal rhabdomyosarcomas using oligonucleotide probes and the polymerase chain reaction. *Cancer Res* **49**: 6324–6327

Wilke W, Maillet M and Robinson R (1993) H-ras-1 point mutations in soft tissue sarcomas. *Mod Pathol* **6**: 129–132

Wurl P, Taubert H, Bache M, Kroll J, Meye A, Berger D, Siermann A, Holzhausen HJ, Hinze R, Schmidt H and Rath FW (1996) Frequent occurrence of p53 mutations in rhabdomyosarcoma and leiomyosarcoma, but not in fibrosarcoma and malignant neural tumors. *Int J Cancer* **69**: 317–323

*© 2000 Cancer Research Campaign*
